# Mast cell activation disrupts interactions between endothelial cells and pericytes during early life allergic asthma

**DOI:** 10.1172/JCI173676

**Published:** 2024-03-15

**Authors:** Régis Joulia, Franz Puttur, Helen Stölting, William J. Traves, Lewis J. Entwistle, Anastasia Voitovich, Minerva Garcia Martín, May Al-Sahaf, Katie Bonner, Elizabeth Scotney, Philip L. Molyneaux, Richard J. Hewitt, Simone A. Walker, Laura Yates, Sejal Saglani, Clare M. Lloyd

**Affiliations:** 1 National Heart and Lung Institute (NHLI), Imperial College London, London, United Kingdom (UK).; 2 Department of Thoracic Surgery, Hammersmith Hospital, London, UK.; 3 Department of Paediatric Respiratory Medicine, Royal Brompton Hospital, London, UK.; 4 Royal Brompton and Harefield Hospitals, Guy’s and St. Thomas’ NHS Foundation Trust, London, UK.

**Keywords:** Inflammation, Vascular biology, Asthma, Mast cells, Pericytes

## Abstract

Allergic asthma generally starts during early life and is linked to substantial tissue remodeling and lung dysfunction. Although angiogenesis is a feature of the disrupted airway, the impact of allergic asthma on the pulmonary microcirculation during early life is unknown. Here, using quantitative imaging in precision-cut lung slices (PCLSs), we report that exposure of neonatal mice to house dust mite (HDM) extract disrupts endothelial cell/pericyte interactions in adventitial areas. Central to the blood vessel structure, the loss of pericyte coverage was driven by mast cell (MC) proteases, such as tryptase, that can induce pericyte retraction and loss of the critical adhesion molecule *N*-cadherin. Furthermore, spatial transcriptomics of pediatric asthmatic endobronchial biopsies suggests intense vascular stress and remodeling linked with increased expression of MC activation pathways in regions enriched in blood vessels. These data provide previously unappreciated insights into the pathophysiology of allergic asthma with potential long-term vascular defects.

## Abstract 2

Mast cell activation during early life allergic asthma leads to the disrupted interaction between endothelial cells and pericytes and reduced vascular functions.

## Graphical abstract

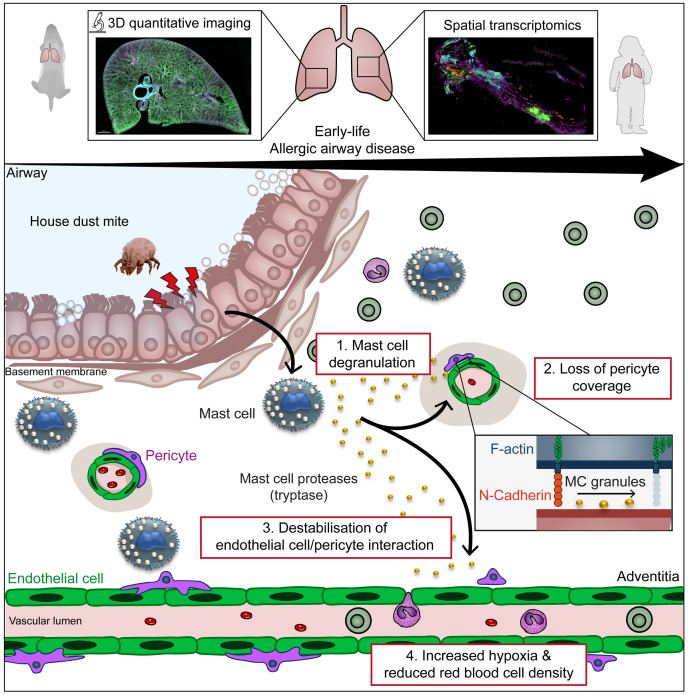

## Introduction

Allergic asthma commonly develops during early childhood. While it is difficult to precisely define the prevalence of children affected, it is now estimated that between 5% and 20% of all children are affected by asthma (1, 2). Chronic inflammation and tissue remodeling are central elements of allergic asthma. Although the immune mechanisms by which allergic responses occur are well characterized in adult pathologies (3, 4), our understanding of the impact of dysregulated immune responses in early life is only now being appreciated. In addition to airway hyperresponsiveness (AHR) and active tissue remodeling, such as increased subepithelial basement membrane thickness, vascular remodeling is also a key feature of allergic asthma (5–7). Indeed, as the disease progresses, the newly altered tissue requires the formation of new blood vessels to efficiently irrigate the remodeled epithelium and adjacent areas (8). The latter has been demonstrated in murine models and human biopsies where an increased number of blood vessels was observed in subepithelial areas (9, 10). Furthermore, most of the intermediate-to-large blood vessels present in the lungs are in close proximity to large epithelial areas described as bronchovascular spaces or adventitia that are involved in multiple pathologies, such as asthma and helminth infections (11–14). Central to the maintenance of blood vessels (15–17), little is known about the interaction between endothelial cells and their associated mural cells, pericytes, during lung pathologies (17–19). Pericytes have a unique ability to maintain endothelial cell fitness through the secretion of proangiogenic mediators, but also cell-cell interaction involving adhesion molecules such as *N*-cadherin (20).

While lung function starts to decline in children with allergic asthma, the lung vasculature is still developing toward its adult form. Therefore, preventing remodeling during early life acts in a critical window of opportunity to prevent disorders later in life. However, the mechanism by which the microcirculation and intermediate blood vessels in close vicinity to large airways are affected by the responses to allergens remains enigmatic. In this context, mast cells (MCs) are key players in orchestrating the development of allergic asthma through the release of preformed mediators (e.g., histamine, proteases) in response to IgE/antigen–mediated or neuropeptide-mediated stimulation (3, 21). However, how MCs can regulate vascular inflammation during early life allergic asthma remains to be determined.

Here, we used our precision-cut lung slice (PCLS) approach, innovative image quantification, functional assays, and spatial transcriptomics to study the lung vasculature in the context of early life allergic airway disease (AAD). Using high-dimensional analyses, we discovered that neonatal mice exposed to allergen exhibit vascular remodeling during disease progression, which is defined by a loss of pericyte coverage, reduced density of red blood cells, and development of hypoxic areas. Mechanistically, we identified a population of adventitial connective tissue MCs (CTMCs) that were highly activated following allergen exposure. In vitro, mouse and human MC granules were able to induce pericyte retraction and reduction of *N*-cadherin expression. The latter was mediated through MC protease activity and specifically, in human MCs, by tryptase. Finally, spatial transcriptomic data of endobronchial biopsies from children pointed out genetic changes in vessel-rich areas associated with cellular stress, remodeling, and MC activation. Together, our data have revealed a new MC/pericyte axis critical in disruption of lung vascular function during early life asthma with potential long-term consequences.

## Results

### Development of a quantitative imaging platform to study lung vasculature in neonatal mice.

**Figure 1:**
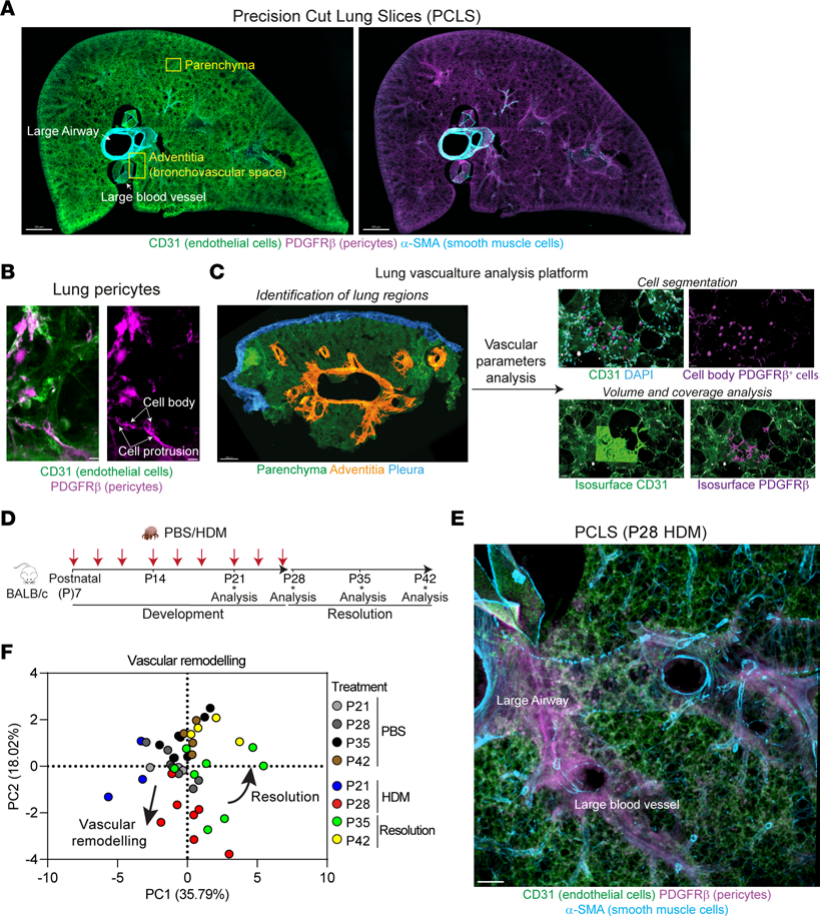
Allergen-induced inflammation leads to vascular remodeling in early life. (**A**) 3D rendering of a PCLS section (200 μm thickness) of neonatal lung (P28) stained for CD31 (green, endothelial cells), α-SMA (cyan, SMCs), and PDGFRβ (magenta, pericytes). Yellow box regions indicate adventitial and parenchyma regions analyzed (see Supplemental Video 1). Scale bars: 500 μm. Representative of 4 independent experiments. (**B**) Zoomed in image of pericytes (PDGFRβ^+^) extending protrusions around endothelial cells. Scale bars: 7 μm. Representative of 4 independent experiments. (**C**) Image analysis pipeline showing the results of cell segmentation and volume analysis. Scale bars: 500 μm (left); 30 μm (right). (**D**) BALB/c mice aged 7 days were exposed to intermittent intranasal PBS or HDM for 3 weeks (red arrows). Lungs were collected at P21, P28, P35, and P42. (**E**) PCLS section of HDM-exposed neonates (P28) showing the vasculature in an adventitial region. Scale bar: 200 μm (representative of 4 independent experiments). (**F**) PCA analysis of lung vascular functions (see Supplemental Figure 2, *n* = 44 mice from 4 independent experiments).

To determine the impact of early life allergic airways disease on the vasculature, we extended our PCLS approach (22) to simultaneously analyze the different components of the vessel wall, namely, endothelial cells and pericytes. Pericytes are a heterogenous population of cells expressing different phenotypic markers between and within organs (20, 23). We explored the Lung Endothelial Cell Atlas (http://lungendothelialcellatlas.com/) data set to identify the most reliable markers of lung pericytes (24). The genes for PDGFRβ and NG2 (*CSPG4*) were the most highly expressed in mouse and human pericytes, and we focused on PDGFRβ due to its greater level of expression in lung pericytes (Supplemental Figure 1, A and B; supplemental material available online with this article; https://doi.org/10.1172/JCI173676DS1). PCLS explants obtained from lungs of neonatal mice were stained for CD31 (endothelial cells), α-SMA (smooth muscle cells [SMCs]), and PDGFRβ (pericytes) and showed a rich and complex network of blood vessels. Interestingly, pericytes were highly abundant in adventitial regions associated with larger blood vessels, but were also present across the parenchyma (Figure 1A and Supplemental Video 1). High-magnification images revealed intimate cellular interactions between endothelial cells and pericytes, with extensive protrusions emerging from the pericyte cell body and surrounding endothelial cells (Figure 1B). In addition, PDGFRβ^+^ cells were also positive for neural/glial antigen 2 (NG2) in adventitial and parenchymal areas, confirming their pericyte phenotype (Supplemental Figure 1B). We next developed an integrative platform to analyze vascular changes using our PCLS system. This platform relies on the use of tile-scan imaging to identify the different lung regions (i.e., bronchovascular space/adventitia and parenchyma) and high-resolution images to define 3D structure and spatial organization of blood vessels. Using Imaris software, we processed the images through cell segmentation and volume analyses. Together, we extracted 6 different parameters of vascular inflammation in both the lung adventitia and parenchyma: (a) endothelial cell coverage (i.e., volume of endothelial cells within the image), (b) vessel density, (c) pericyte number, (d) pericyte coverage (i.e., volume of pericytes surrounding endothelial cells), (e) distance between endothelial cell and pericyte, and (f) number of CD45^+^ cells (Figure 1C). Together, these parameters provide an accurate representation of the lung vascular architecture and its impact on inflammation.

Next, we employed our platform to analyze the lung vasculature in neonatal mice exposed to house dust mite (HDM) (25). Seven-day old BALB/c mice were exposed to intermittent HDM, and lung lobes were collected at different times during disease progression (i.e., P21 and P28) and during the resolution phase after the end of the exposure (i.e., P35 and P42; Figure 1, D and E). As we previously reported for this model, HDM induces all the features of allergic asthma, including increased airway resistance and strong recruitment of eosinophils, T cells, and innate lymphoid cells 2 (ILC2s) in the airways (25). We performed an unsupervised principal component analysis (PCA) combining the 6 vascular parameters extracted from our images in 2 regions of the lungs (i.e., adventitia and parenchyma). Adventitial regions were identified using structural parameters (i.e., presence of a large airway and intermediate/large blood vessel), and the associated microvasculature within a 150 μm radius from the large airway and large vessel was analyzed. Parenchyma was defined using alveolar structures far from any large airway (Figure 1A). While some individual parameters were not significantly different between groups, we did see effects in the total variance when inspecting the principal components of these 12, not independent, parameters. The PCA revealed alterations to the vascular structure as early as 2 weeks following HDM exposure (P21). These changes were further exaggerated by the end of the allergen exposure (P28) and were mainly linked to a loss of the microcirculation. While some mice still exhibited the same vascular changes at P35 during the resolution phase, most had recovered a vasculature comparable to that of age-matched controls at P42 (Figure 1F; loadings, eigenvalues, and individual data points shown in Supplemental Figure 2, A–K, and Supplemental Figure 4B).

In summary, our data clearly indicate that HDM exposure induced vascular remodeling in early life mainly due to vascular loss. These changes are maintained during the resolution phase, but slowly resolve with time.

### Loss of pericyte coverage occurs early in HDM-exposed neonatal mice.

**Figure 2:**
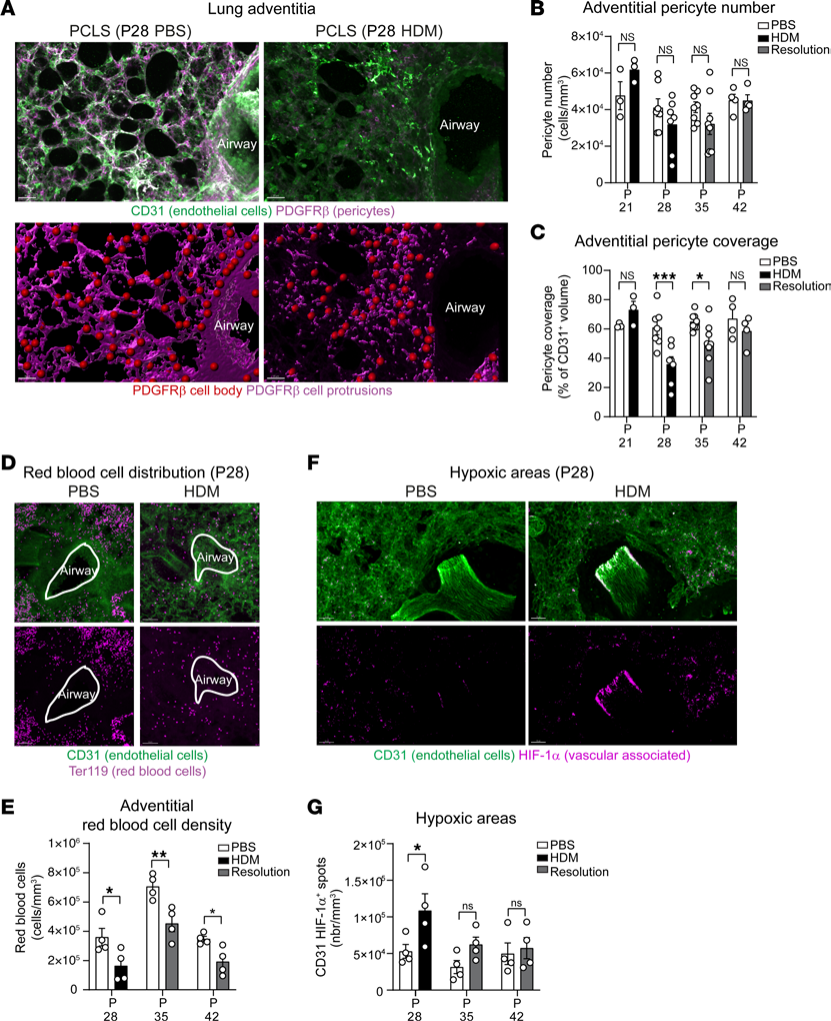
Repeated HDM exposure leads to loss of pericyte protrusions, reduced red blood cells, and hypoxic areas. Neonate mice were exposed with PBS or HDM as indicated in Figure 1D. (**A**) 3D rendering of a PCLS section in PBS- and HDM-exposed mice 3 weeks after first inhalation showing CD31 (green, endothelial cells) and PDGFRβ (magenta, pericytes). Lower panels show pericyte cell body (red dots) and protrusion (magenta surface) analyses (see Supplemental Video 2). Scale bars: 30 μm (representative of 4 independent experiments). (**B** and **C**) PDGFRβ^+^ pericyte number per mm^3^ (**B**) and coverage (**C**, normalized to the total volume of CD31^+^ blood vessel) in the lung adventitia. *n* = 3–8 mice per group from 4 independent experiments. (**D**) Representative PCLS showing reduced red blood cell (Ter119^+^, purple) density in the microcirculation (CD31, green). Scale bars: 50 μm. Representative of 3 independent experiments. (**E**) Adventitial red blood cell density normalized to the total volume of the image. *n* = 3–4 mice per group from 3 independent experiments. (**F**) PCLS of HDM-exposed mice for 3 weeks exhibiting increased HIF-1α (purple) associated to the vasculature (CD31, green). Scale bars: 30 μm. Representative of 4 independent experiments. (**G**) Number of HIF-1α spots in adventitial region in PBS- and HDM-exposed mice (*n* = 4 mice per group). Data are represented as means ± SEM. *P < 0.05; **P < 0.01;***P < 0.001, 2-way ANOVA followed by Šidák’s post hoc test.

One of the principal parameters determining the vascular remodeling in our PCA analysis (Figure 1F) was the change in pericyte coverage in close proximity to the lung adventitia. Pericyte coverage, or the volume of pericyte cell protrusions, is an essential factor for maintaining endothelial cell fitness and overall blood vessel structure, such as in the brain where pericytes form the blood-brain barrier (15). Zoomed in images clearly indicate that, during early life AAD, while the endothelial cell structure was still present in close vicinity to large airways at P28, the pericyte signal was greatly reduced (Figure 2A and Supplemental Video 2). Interestingly, our image analysis revealed that pericyte cell bodies were still present in PBS and HDM groups, but the extent of pericyte protrusions was severely reduced following 3 weeks of HDM exposure (Figure 2A). Indeed, quantitative analysis confirmed that adventitial pericyte cell number was not significantly changed during mouse development or affected by HDM exposure (Figure 2B). In contrast, pericyte coverage was reduced after 3 weeks of allergen exposure (i.e., approximately 42% reduction) and slowly recovered in the resolution phase to nearly physiological levels 2 weeks after last HDM inhalation (Figure 2C). Endothelial cell volume or blood vessel density did not show strong differences; however, we detected a small reduction in endothelial cell volume at the end of the HDM exposure and this decrease was significant 1 week into the resolution phase (Supplemental Figure 2E). The latter may indicate that the reduction in pericyte coverage leads to a decrease in the microcirculation of lung adventitial regions. Finally, we assessed the functional consequences of the adventitial vascular changes. To this end, we analyzed the distribution of red blood cells and the expression of HIF-1α in the vasculature (i.e., CD31^+^ areas). Following 3 weeks of HDM exposure, the adventitial vasculature showed reduced red blood cell density (Figure 2, D and E) and increased expression of HIF-1α (Figure 2, F and G). Interestingly, while HIF-1α slowly came back to normal in the resolution phase (Figure 2G), the red blood cell density reduction was still present 2 weeks after the end of the challenge (Figure 2E). To investigate the long-term consequences of early life AAD, we treated neonates for 3 weeks with HDM and reexposed mice to a single dose of allergen or PBS at P42, 2 weeks after the last HDM exposure (Supplemental Figure 3A). While the mice retreated with HDM did not exhibit a reduction in pericyte number compared with control animals, pericyte coverage was significantly reduced (~26% reduction, Supplemental Figure 3, B–D). In addition, we did not observe changes in other vascular parameters in the lung adventitia (i.e., distance endothelial cell/pericyte, endothelial cell volume, and blood vessel density), but an increased expression of HIF-1α indicating local change in oxygen concentration (Supplemental Figure 3, E–I). These data clearly indicate that allergen exposure during early life leads to functional consequences on the vasculature that can be long lasting.

Collectively, these data demonstrate, for the first time to our knowledge, that repeated exposure to allergen during the early life developmental period induces loss of pulmonary pericyte protrusions and blood vessels, leading to hypoxic areas in the pulmonary microcirculation.

### Lung adventitia is marked by immune cell infiltration and MC activation.

**Figure 3:**
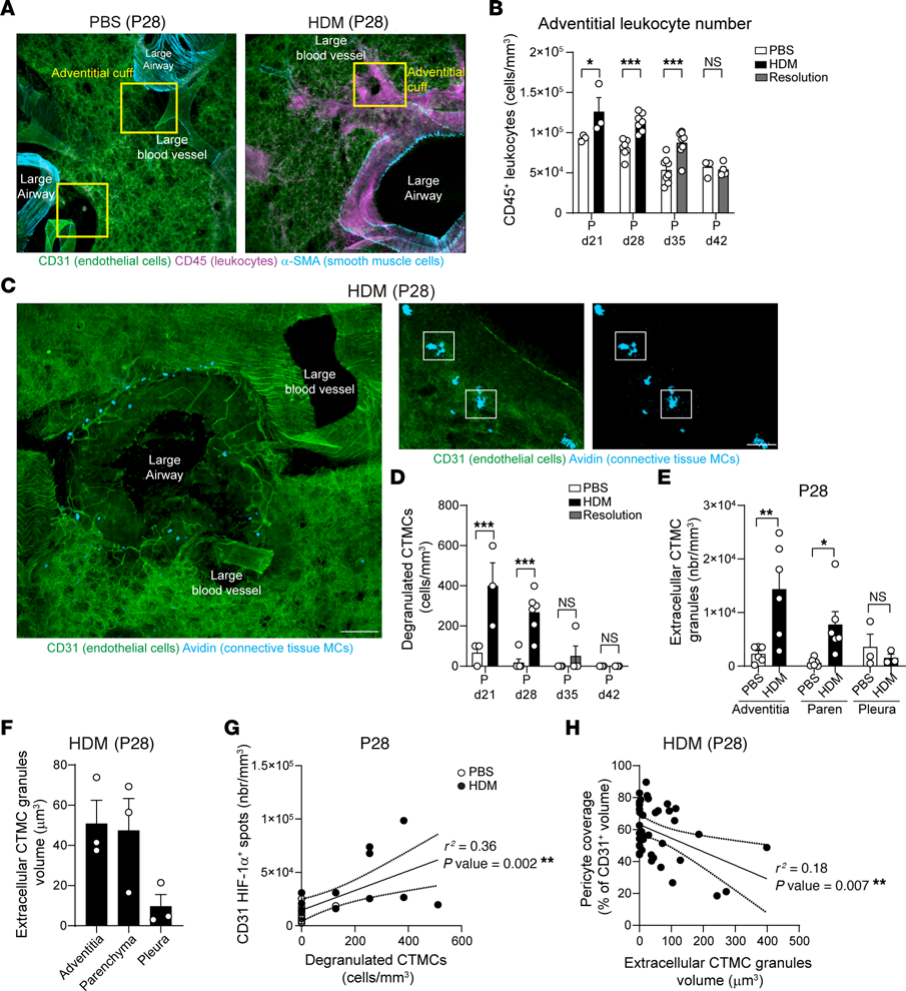
Early life allergen exposure leads to immune-cell recruitment and MC activation in the lung adventitia. (**A**) 3D rendering of a PCLS in the lung adventitia in PBS- and HDM-exposed mice at P28 showing CD31 (green, endothelial cells), α-SMA (blue, SMCs), and CD45 (magenta, leukocytes). Scale bars: 200 μm. Representative of 4 independent experiments. (**B**) CD45^+^ cell number. *n* = 3–8 mice per group from 4 independent experiments. (**C**) Representative 3D image of lung adventitia showing the distribution of CTMCs (avidin, blue) around a large airway and associated vasculature (CD31, green) and images showing degranulated MCs adjacent to large airways and blood vessels (see Supplemental Video 3). Scale bars: 150 μm (left); 50 μm (right). Representative of 4 independent experiments. (**D**) Number of degranulated MCs. *n* = 3–6 mice per group. (**E** and **F**) Number of extracellular CTMC granules per mm^3^ (**E**) and volume (**F**). *n* = 3–6 mice per group from 3 independent experiments. (**G**) Correlation between vascular-associated HIF-1α^+^ and number of degranulated CTMC granules. *n* = 23 images from 4 PBS- and 4 HDM-treated mice. (**H**) Correlation between pericyte coverage and volume of extracellular CTMC granules. *n* = 39 images from 4 PBS- and 4 HDM-treated mice. Data are represented as means ± SEM. *P < 0.05; **P < 0.01;***P < 0.001, 2-way ANOVA followed by Šidák’s post hoc test (**B**, **D**, and **E**); Spearman’s rank correlation test (**G** and **H**).

Lung adventitial regions are characterized by an abundant presence of resident immune cells coupled with intense recruitment of various leukocytes during inflammation (14, 22, 26). We hypothesized that local activation of the immune system may play a role in the vascular remodeling and changes in pericyte morphology observed in our neonatal mice following inhaled HDM exposure. Therefore, we employed our image analysis platform to dissect immune cell recruitment and activation following HDM exposure (Figure 3A) in the lung adventitia and parenchyma. We observed a significant increase in the number of adventitial CD45^+^ leukocytes following each HDM exposure (i.e., approximately 36% and approximately 40% increase after 2 and 3 weeks of HDM, respectively), with the difference still present 1 week into the resolution phase (i.e., approximately 60% increased) and returning to physiological levels 2 weeks following the end of the allergen-exposure period (Figure 3, A and B). Immune cell recruitment was mainly restricted to the periadventitial regions, since the lung parenchyma did not show a significant increase in leukocytes (Supplemental Figure 4, A and B).

Given that MCs play a key role in allergic asthma and that we recently demonstrated the impact of perivascular CTMCs in regulating pericyte function (27), we hypothesized that MCs could play a role in mediating pericyte change during early life AAD. We utilized fluorescent avidin staining to analyze the profile of degranulated CTMCs, as we and others have demonstrated that it provides an accurate measurement of the localization and cellular interactions of extracellular CTMC granules (28–31). While the number of CTMCs did not change in HDM-exposed neonatal mice (Supplemental Figure 4C), we observed a clear increase in the number of degranulated CTMCs every time mice were challenged with allergen (Figure 3, C and D, and Supplemental Video 3). We found extracellular CTMC granules were abundant in the lung adventitia and within the parenchyma, but not in the lung pleural cavity (Figure 3E). These granules retain a large volume (i.e., approximately 50 μm^3^) even outside of the MC body, and no differences were observed between adventitial and parenchymal MC granule volume (Figure 3F and Supplemental Video 3). Interestingly, we observed a positive correlation between the extent of degranulated CTMCs and expression of HIF-1α (*r^2^* = 0.36, *P* = 0.002, Figure 3G). Furthermore, the volume of CTMC granules was negatively correlated with the reduction in pericyte coverage (*r^2^* = 0.18, *P* = 0.007, Figure 3H), indicating that regions with large extracellular CTMC granules are associated with changes in pericyte morphology. Finally, the presence of CTMC granules was linked with an increased distance between endothelial cells and pericytes (Supplemental Figure 4D).

In summary, our data show that upon exposure to allergen, neonatal mice develop strong adventitial inflammation linked with activation and degranulation of resident CTMCs. Areas with large CTMC granules are associated with destabilization of endothelial cell/pericyte interaction that likely disrupt the vasculature, leading to hypoxic areas.

### CTMC degranulation induces pericyte retraction and loss of N-cadherin.

**Figure 4:**
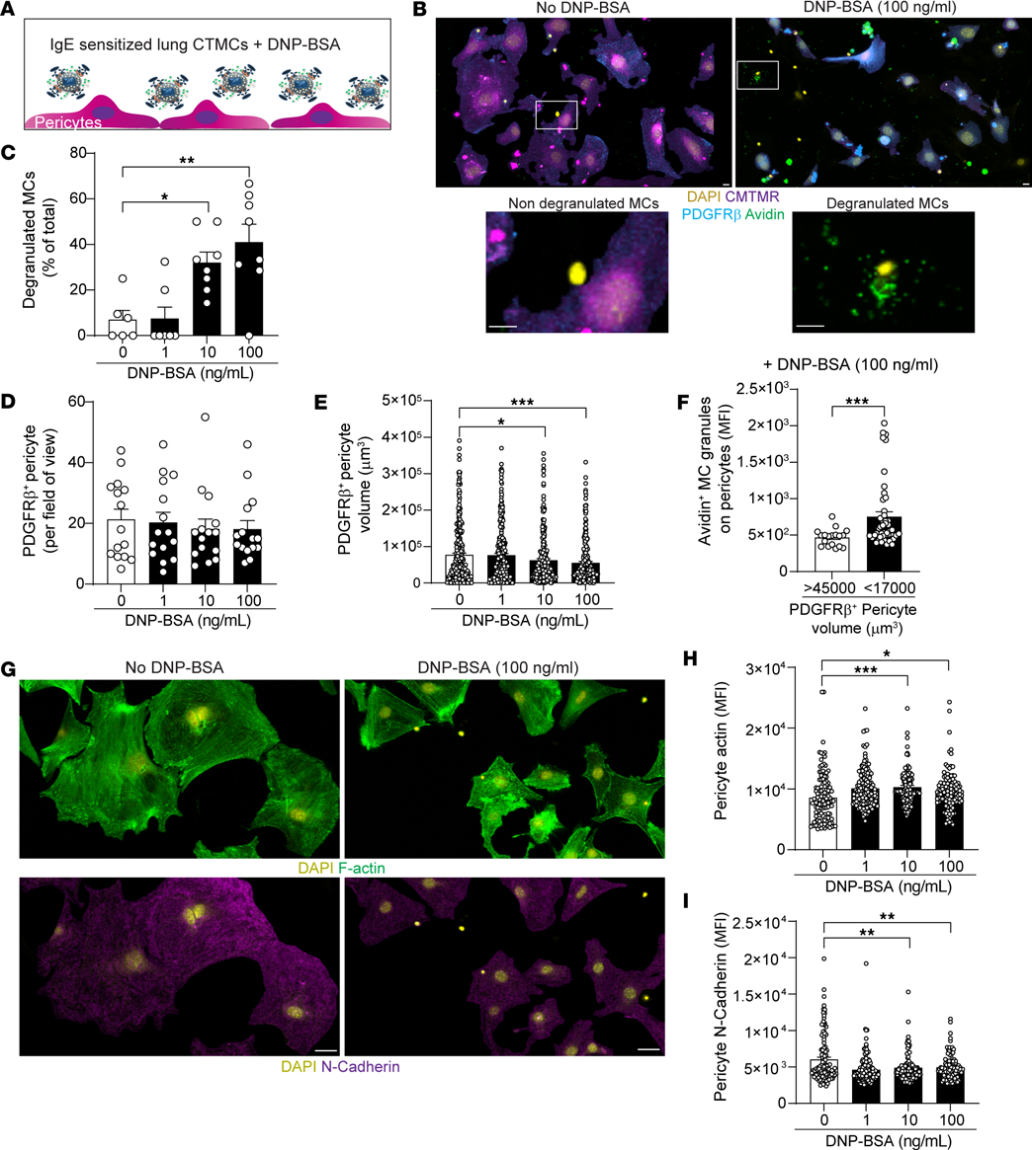
MC granules induce pericyte retraction and cleavage of surface *N*-cadherin. MCs were sensitized overnight with anti-DNP IgE then placed on a layer of pericytes (stained with CMTMR) and stimulated with increasing concentrations of DNP-BSA for 24 hours. (**A**) Schematic depicting the coculture experiment between primary mouse lung MCs and pericytes. (**B**) Images of unstimulated or stimulated (100 ng/ml DNP-BSA) pericyte/MC cocultures stained for DAPI (yellow), CMTMR (purple), PDGFRβ (blue), and avidin (green, MC granules). White boxes indicate the areas zoomed in showing examples of resting or degranulated MCs. Scale bars: 15 μm. Representative of 3 independent experiments. (**C**) Frequency of degranulated MCs. *n* = 6–8 images from 3 independent experiments. (**D**) Number of pericytes per field of view. *n* = 15 images from 3 independent experiments. (**E**) Pericyte volume determined using the cell tracer CMTMR. *n* = 253–299 pericytes from 3 independent experiments. (**F**) Avidin signal on small pericytes (<17,000 μm^3^) and large pericytes (>45,000 μm^3^) showing that small pericytes exhibit more MC granule staining on their surfaces. *n* = 18–46 from 3 independent experiments. (**G**) Representative images of F-actin (green) and *N*-cadherin (magenta) pericyte expression in the presence of degranulated MCs or control. Scale bars: 50 μm. Representative of 3 independent experiments. (**H**–**I**) F-actin (**H**, *n* = 98–144) and surface *N*-cadherin (**I**, *n* = 98–144) MFI on pericytes. Three independent experiments. Data are represented as means ± SEM. *P < 0.05; **P < 0.01; ***P < 0.001, 1-way ANOVA followed by Tukey’s post hoc test (**C**, **D**, **E**, **H**, and **I**); 2-tailed Student’s *t* test (**F**).

To analyze the functional impact of CTMC degranulation on lung pericytes, we developed an in vitro coculture model between primary lung MCs and pericytes. Lung cells were purified from naive mouse lungs, and following 2 to 4 weeks in culture, a high level of purity was detected for both cell populations with expression of characteristic markers: NG2^+^/PDGFRβ^+^ and FcεRI^+^/ST2^+^/CD117^+^ for pericytes and MCs, respectively (Supplemental Figure 5A). In addition, lung MCs were positive for avidin signal, indicating that the in vitro–generated MCs were indeed CTMCs (Supplemental Figure 5A). Anti-dinitrophenyl (anti-DNP) IgE-sensitized lung MCs were added to a layer of fluorescently labeled pericytes (CMTMR^+^) and stimulated with increasing concentrations of DPN-BSA to induce FcεRI crosslinking and degranulation (Figure 4A). Degranulated lung MCs were identified using extracellular avidin staining, revealing the presence of exteriorized granules in the surrounding environment or still associated with the surface of MCs (Figure 4B). While the frequency of degranulated MCs increased proportionally with the concentration of DNP-BSA (Figure 4C), the number of pericytes was not affected by MC activation status (Figure 4, B and D). However, as observed in vivo, the volume of pericytes decreased significantly 24 hours after stimulation (approximately 30% reduction for 100 ng/ml DNP-BSA; Figure 4, B and E). We next investigated whether the reduction in volume was more pronounced in pericytes that were directly in contact with MC granules. Avidin MFI on the largest pericytes (i.e., >45000 μm^3^) showed lower avidin signal compared with the smallest pericytes (i.e., <17000 μm^3^), indicating that the smallest pericytes had more MC granules on their surfaces that could induce their retraction (Figure 4F). Furthermore, one of the main consequences of pericyte retraction is the polarization of F-actin (32). Indeed, concomitant with reduced pericyte volume, the increased number of degranulated MCs led to an enhanced intracellular F-actin signal per unit of volume within pericytes (Figure 4, G and H). Interestingly, expression of *N*-cadherin, one of the main cadherins involved in the interaction between endothelial cells and pericytes (33), was decreased following contact with MC granules (approximate 20% reduction at 100 ng/ml DNP-BSA, Figure 4I). Collectively, MC granules can efficiently induce pericyte retraction in a dose-dependent manner and facilitate reduction in *N*-cadherin expression.

### Pericyte retraction and N-cadherin loss are mediated by MC proteases.

**Figure 5:**
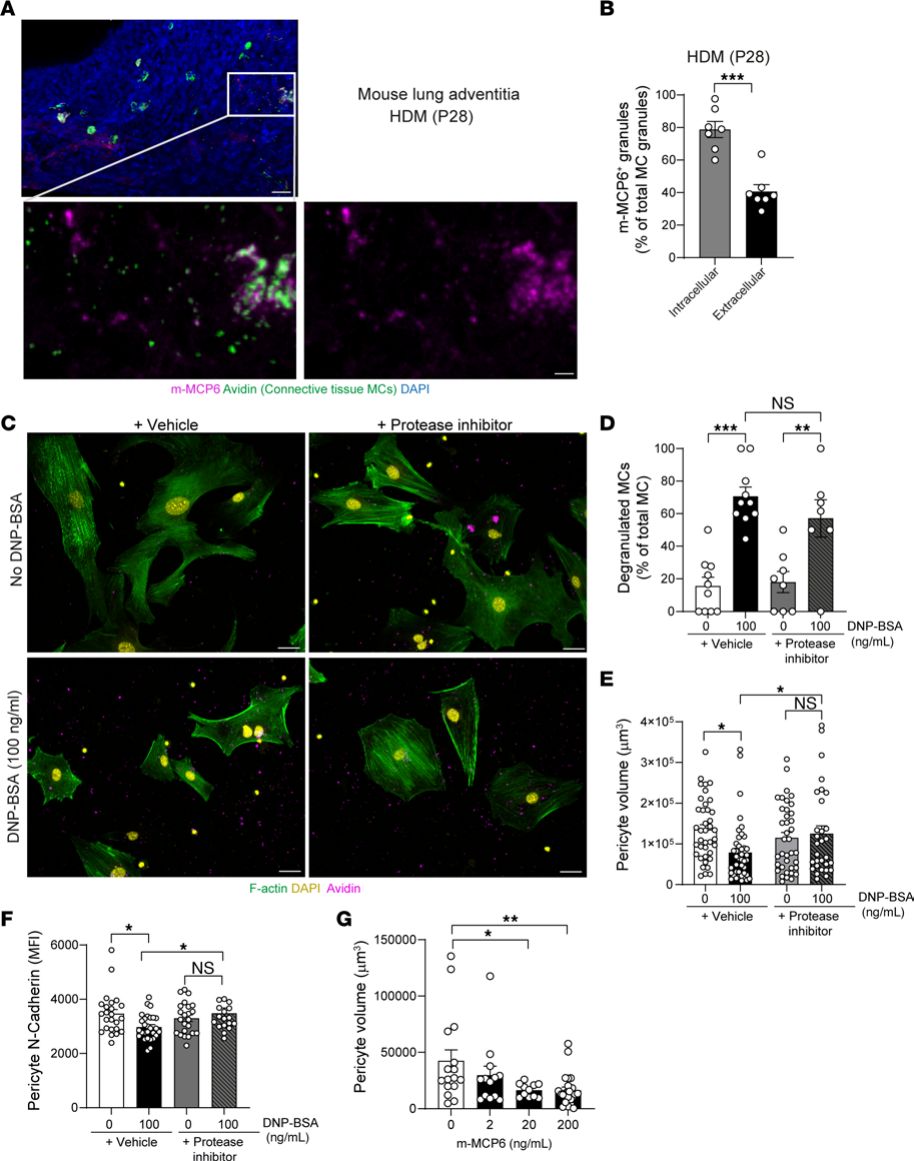
MC-derived proteases induce pericyte retraction and *N*-cadherin cleavage. (**A** and **B**) Neonate mice were exposed to HDM for 3 weeks. (**A**) 3D rendering of a PCLS section in the lung adventitia in HDM-exposed mice showing DAPI (blue), m-MCP6 (mouse tryptase, magenta), and MCs (avidin, green); lower panels show zoomed-in images of the white box region and the m-MCP6 signal in extracellular MC granules. Scale bars: 30 μm (upper panel); 10 μm (lower panels). (**B**) Colocalization analyses between intracellular and extracellular MC granules (avidin^+^) and m-MCP6 showing frequency of m-MCP6^+^ granules. Each dot represents an image from 3 independent mice. (**C**–**F**) MCs were sensitized overnight with anti-DNP IgE, then placed on a layer of pericytes (stained with CMTMR) and stimulated with increasing concentrations of DNP-BSA for 24 hours in the presence of a protease inhibitor cocktail or vehicle control (DMSO). (**C**) Images of unstimulated or stimulated (100 ng/ml DNP-BSA) pericyte/MC cocultures stained for DAPI (yellow), F-actin (green), and MC granules (avidin, magenta). Scale bars: 50 μm. Representative of 3 independent experiments. (**D**) Number of degranulated MCs normalized to the total number of MCs. Each dot represents an image from 3 independent experiments. (**E**) Pericyte volume and (**F**) cell-surface *N*-cadherin MFI on pericytes. Each dot represents an individual pericyte from 3 independent experiments. (**G**) Lung pericyte volume 24 hours following recombinant m-MCP6 exposure. Each dot represents an individual pericyte from 3 independent donors. Data are represented as means ± SEM. *P < 0.05; **P < 0.01; ***P < 0.001, 2-tailed Student’s *t* test (**B**); 1-way ANOVA followed by Tukey’s post hoc test (**D**, **E**, **F**, and **G**).

Having observed reduced pericyte volume and *N*-cadherin expression on pericytes following MC degranulation, we next investigated the molecular mechanisms of MC-induced pericyte retraction. MCs contain a vast repertoire of proteases, which are involved in protective immunity against venoms and parasites, but can also be detrimental in the context of inflammatory pathologies such as asthma (34, 35). We hypothesized that MC-derived proteases could be involved in pericyte retraction. Indeed, PCLS of HDM-exposed neonates (P28) indicated that proteases such as m-MCP6 (MC tryptase) were present in CTMC granules (Figure 5A). Furthermore, detailed colocalization analysis indicated the presence of m-MCP6 in both intracellular and extracellular MC granules, 24 hours after last HDM challenge, as demonstrated by their overlap with avidin^+^ regions (Figure 5B). The latter agrees with the requirement for m-MCP6 to be associated with the CTMC granule matrix to be stored and activated efficiently (35, 36). We next tested to determine whether a general protease inhibitor could prevent pericyte protrusion loss in the presence of degranulated MCs. Interestingly, our results showed that, although MC degranulation was not affected by the protease inhibitor (Figure 5, C and D), pericyte volume and *N*-cadherin surface expression were maintained (Figure 5, E and F). To further confirm this observation, we directly exposed lung pericytes to recombinant m-MCP6 and observed an approximately 62% reduction in pericyte volume, indicating that m-MCP6 at a high concentration is sufficient to induce pericyte retraction (Figure 5G). Together, these data demonstrate that MC proteases can efficiently induce pericyte retraction and loss-of-surface *N*-cadherin and potentially lead to loss of the endothelial cell/pericyte interaction.

### Spatial transcriptomic analysis suggested cellular stress in vessel-rich areas of children with asthma and human lung pericytes retract following MC degranulation.

**Figure 6:**
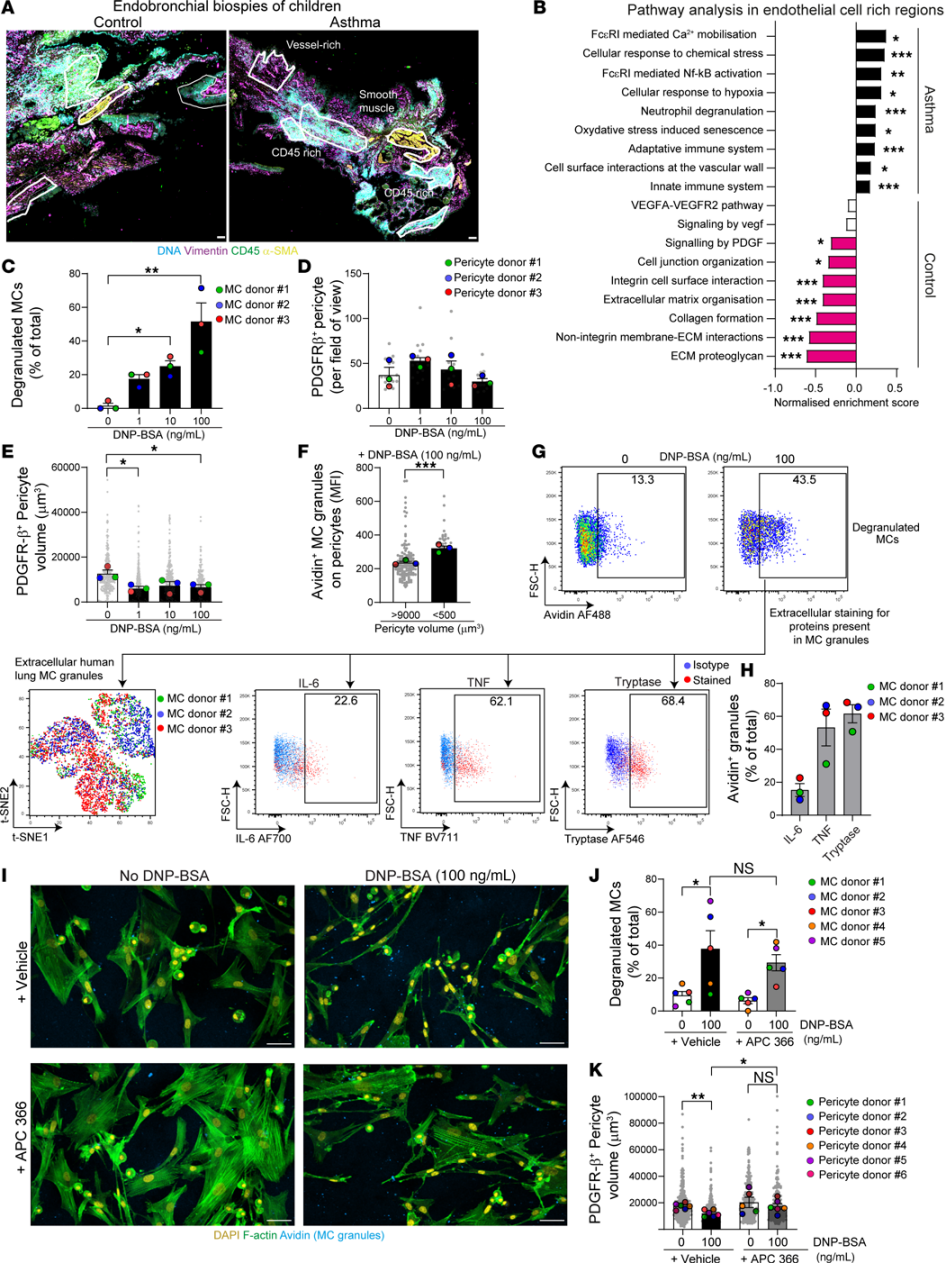
Transcriptional signature of children with asthma suggests vascular stress, and human MCs induce tryptase-dependent pericyte retraction. (**A**) Immunofluorescence images of endobronchial biopsies stained for DNA (Syto83, green), vimentin (purple), CD45 (blue), and α-SMA (yellow) showing the selected ROIs (boxed regions). Scale bars: 100 μm. (**B**) Pathway enrichment analysis in endothelial cell–rich regions of children with asthma and controls. *n* = 4–9 ROIs per group from 2 controls and 2 with asthma. *P* values in Supplemental Table 4. (**C**–**F** and **I**–**K**) Human MCs were sensitized overnight with anti-DNP IgE, then placed on pericytes and stimulated with an increasing concentration of DNP-BSA for 24 hours; (**I**–**K**) APC366 (tryptase inhibitor) or vehicle control was added at the time of stimulation. (**C**) Degranulated MC number normalized to the total number of MCs. *n* = 3 MC donors from 3 independent experiments. (**D**) Pericyte number. *n* = 3 pericyte donors from 3 independent experiments. (**E**) Pericyte volume. *n* = 3 pericyte donors from 3 independent experiments. (**F**) Avidin signal on small pericytes (<500 μm^3^) and large pericytes (>9,000 μm^3^). *n* = 3 pericyte donors from 3 independent experiments. (**G**) Flow cytometry profiles of degranulated MCs and unsupervised analysis of extracellular MC granules. t-SNE analysis performed on 3 pooled donors. Representative of 2 independent experiments. (**H**) Frequency of avidin^+^ granules positive for indicated markers. *n* = 3 MC donors from 2 independent experiments. (**I**) Images of pericyte/MC coculture stained for DAPI (yellow), F-actin (green), and MC granules (avidin, blue). Scale bars: 50 μm. Representative of 3 independent experiments. (**J**) Degranulated MC numbers normalized to the total number of MCs. *n* = 5 MC donors from 3 independent experiments. (**K**) Pericyte volume. *n* = 6 pericyte donors from 3 independent experiments. Data are represented as means ± SEM. *P < 0.05; **P < 0.01;***P < 0.001, 2-tailed Mann-Whitney test (**B**); 1-way ANOVA followed by Tukey’s post hoc test (**C**–**E**); 2-tailed Student’s *t* test (**F**); 2-way ANOVA followed by Šidák’s post hoc test (**J** and **K**).

We next employed digital spatial profiling with the NanoString GeoMx Cancer Transcriptome Atlas (CTA, approximately 1,800 genes) to analyze vascular remodeling and MC activation in 4 children with severe asthma (between 9 and 17 years old; Supplemental Table 1) and 2 controls (between 8 and 11 years old; Supplemental Table 1). Formalin-fixed paraffin-embedded (FFPE) sections obtained from endobronchial biopsies were stained for DNA, vimentin, CD45, and α-SMA to determine 4 types of regions, namely, epithelium (defined using morphology and DNA stain), immune cell infiltrate (CD45^+^ rich), smooth muscle, and fibroblast-rich areas (Figure 6A) (37).

We analyzed 40 regions of interest (ROIs) (Supplemental Table 2) and first defined the regions with the highest density of blood vessels using endothelial cell and pericyte cell deconvolution using single cell RNA-Seq (scRNA-Seq) cell-type signatures (38) (genes employed are indicated in Supplemental Table 3). We selected 16 ROIs from 2 controls and 2 patients with asthma with high endothelial cell signature (i.e., enrichment score >0.15) in fibroblast and immune cell infiltrate areas (Supplemental Figure 6A). While the pericyte enrichment score was not statistically different across the 4 types of regions (Supplemental Figure 6B), we observed a correlation between regions with high endothelial cell and pericyte signatures, suggesting that these regions are enriched in microvessels (Supplemental Figure 6C). The high endothelial cell signature was further suggested by the higher expression of key vascular genes (i.e., *CDH5*, *PECAM1*, *PDGFRB*) in the 16 ROIs identified (Supplemental Figure 6D). Gene set enrichment analysis (GSEA) was employed to analyze pathways differentially regulated between control and asthma ROIs in endothelial cell–rich regions. Although this analysis is limited by the number of ROIs available, patients with asthma had increased signals in pathways related to cellular stress and hypoxia and downregulation of pathways linked to extracellular matrix organization and PDGF signaling (Figure 6B; uncorrected and corrected *P* values are indicated in Supplemental Table 4). Individual gene values for critical pathways (e.g., cellular response to chemical stress, cellular response to hypoxia, signaling by PDGF, and extracellular matrix organization) are shown in Supplemental Figure 6E. Of note, this preliminary analysis suggested no differences in endothelial cell and pericyte enrichment between control and asthma ROIs (Supplemental Figure 6F). Interestingly, MC activation genes such as FcεRI activation pathway were particularly enriched in endothelial cell regions of patients with asthma (Figure 6B). Cell deconvolution analysis hinted that there were a high proportion of MCs in fibroblast and CD45-rich areas (Supplemental Figure 6G) that correlated with the endothelial cell enrichment score (Supplemental Figure 6H). The increased proportion of MCs in endothelial cell–rich regions was further suggested by an increase in expression of the tryptase gene (i.e., *TPSAB1*, Supplemental Figure 6I), and no differences in abundance were observed between patient groups in endothelial cell–rich regions (Supplemental Figure 6J).

Finally, we isolated human pericytes and MCs from healthy lung tissue and performed coculture experiments. Isolation and culture methods yielded purities of more than 95% for both cell types (Supplemental Figure 5B). We exposed lung pericytes to IgE-sensitized MCs and increasing concentrations of DNP-BSA for 24 hours, leading to MC degranulation without affecting pericyte viability (Figure 6, C and D). As observed for murine cells, human pericytes retract following MC activation (Figure 6E) and the volume reduction was enhanced in cells in close contact with MC granules (Figure 6F). To get a better insight into the molecular mechanism of MC-dependent pericyte retraction, we analyzed the content of extracellular MC granules by flow cytometry. Following 30 minutes of IgE-mediated stimulation, we performed an unsupervised analysis (i.e., t-distributed stochastic neighbor embedding [t-SNE]) of membrane-bound MC granules (i.e., avidin^+^ MC; ref. 31), pooling 3 independent stimulated lung MC donors (Figure 6G). We discovered strong heterogeneity in the profile of extracellular MC granules with the presence of cytokines such as IL-6 and TNF and an abundant presence of tryptase in approximately 61.6% of extracellular MC granules (Figure 6H). Since tryptase is highly expressed in extracellular MC granules, we hypothesized that this protease could directly induce pericyte retraction. Therefore, we performed coculture experiments between activated MCs and pericytes in the presence of a specific tryptase inhibitor (i.e., APC-366) (31, 39). While APC-366 did not affect MC degranulation (Figure 6, I and J), pericyte volume was maintained in the presence of the inhibitor (Figure 6, I and K). Overall, our data indicate that tryptase released from human MCs induces loss of pericyte protrusions.

## Discussion

AADs disproportionally affect children at a stage of their lives when the lungs are still developing structurally — including the vast vascular network. Despite our growing understanding of the distinctions between adult and neonatal immune responses (25, 40, 41), the impact of respiratory diseases such as allergic asthma on the lung vasculature remains elusive. Tissue remodeling, such as increased thickness of the reticular basement membrane and collagen deposition, is a key feature of chronic inflammation, and angiogenesis is classically associated with this phenomenon (7, 25, 42). Indeed, as the tissue adjacent to the airway is expanding, newly developed bronchial blood vessels are required to supply the necessary nutrients. Paradoxically, how the original pulmonary microcirculation in adventitial regions adapts to the newly developed tissue currently remains unclear. Here, we demonstrate that pulmonary exposure to inhaled allergens such as HDM during early life drives important vascular changes in the lung adventitia and bronchovascular space. For what we believe is the first time, we provide an accurate characterization of the cascade of events leading to vascular remodeling, starting with a reduction in pericyte coverage, associated with reduced red blood cell density concomitant with increased hypoxic regions in specific lung regions. We show that the loss of protrusions from the pericyte surface is driven by proteases released following MC degranulation. Finally, spatial transcriptomics analysis pointed out a strong remodeling signature in vessel-rich areas of lungs from children with asthma associated with increased MC activation that can induce human pericyte retraction.

Endothelial cell–pericyte interaction is an essential component of the stability of blood vessels; however, the mechanisms by which this interaction is dysregulated during inflammation remain poorly understood (43). Here, we showed that, while the number of pericytes did not change dramatically throughout postnatal lung development or following exposure to allergens, the reduction in pericyte coverage was one of the earliest events observed for the vasculature during the development of AAD. The role of pericytes in lung biology is still poorly understood; fate mapping and depletion models provided the first evidence for the involvement of pericytes in lung pathologies, such as pulmonary hypertension and lung fibrosis (44, 45). Our data concur with previous work showing that in adult mice, the disruption of the PDGFβ/PDGFRβ axis worsened lung function, such as airway hyperreactivity (18). The reduction in pericyte coverage is reminiscent of other pathologies, such as Alzheimer’s disease or following stroke. The latter drives a reduction in blood flow in specific areas, leading to a decline in brain function (46, 47). Mechanistically, we show that mouse and human MC-derived granules induce pericyte retraction. Pericytes respond to a variety of inflammatory stimuli, such as ROS, prostaglandin E2, and TNF (43); however, to our knowledge, the impact of MCs on pericyte coverage has never been described before. We showed that MC-derived proteases such as tryptase induce pericyte retraction and loss of *N*-cadherin. The latter is a crucial junctional molecule mediating endothelial cell–pericyte interactions (48), and further studies are required to understand how MC proteases can cleave *N*-cadherin. In addition, other proteases, such as neutrophil-derived proteases present in neutrophil extracellular traps (NETs) (49), may play a role in pericyte damage that remains to be investigated. The physiological relevance and mechanism by which pericyte retraction occurs are still not fully elucidated. The nature of this phenomenon is difficult to analyze in vivo, and in vitro experiments may not accurately represent how pericytes contract in vivo. Recent developments to provide better markers for pericytes (50) and improved in vitro models will enhance our understanding of this enigmatic cell.

Lung adventitial regions have recently received great interest, and spatial analysis has started to uncover their specialized immune regulation (13, 14). In this context, we showed that lung adventitial regions were central to the inflammatory and remodeling response during early life AAD. Indeed, most of the vascular changes and inflammatory cell recruitment observed were confined to this region, confirming the essential role of spatial approaches to analyzing lung immune responses. However, the limit of the lung adventitia, identity of pericytes in these regions, and whether there is a subclass of pericytes operating during development that are more susceptible to inflammation remain to be determined (51). Furthermore, the complexity of human airways is not perfectly reflected in murine models, and additional work is necessary to provide a better characterization of human adventitial areas.

We identified a population of CTMCs present around the airway and large blood vessels in the neonatal developing lung. This agrees with previous studies that showed an increase in MC numbers from day 7 after birth (40). Our data extend this observation by determining the cellular location of MCs and explore their functional involvement during AAD. The specific distribution of MCs in these regions during early life remains unclear, but the presence of mural cells, such as vascular smooth muscle or subtype of pericytes, could lead to the development of MCs (52). In addition, IL-33 is a potent MC activator able to regulate the extent of MC degranulation and production of chemokines (53). However, despite evidence that the mechanisms of release of IL-33 between mouse and human are different, it is established that IL-33 is critical for immune responses during early life (42, 54). The precise impact of IL-33 on MC activation during early life remains to be explored. Here, we employed IgE-mediated MC stimulation to analyze the impact of MC proteases on pericytes in vitro. While we cannot rule out the involvement of other MC stimuli such as substance P, HDM-treated neonatal mice mount efficient IgE responses following 3 weeks of allergen exposure (54, 55), thus validating our in vitro approach.

Interestingly, our preliminary spatial transcriptomic analysis of endobronchial biopsies from children with asthma suggests a change in the tissue dynamic present during postnatal development. These differences highlight the importance of topographical information as to cell location during health and disease (51). Key open questions remain as to whether these clusters are driven by the immune response to allergens and whether they are maintained during progression to adulthood. As with all early life studies in humans, we are limited here by the lack of healthy controls and restricted to disease control subjects (56). However, although the pathway analysis implies some differences exist, this finding requires further analysis, since the genes present in these pathways are from publicly available gene lists and thus may not provide a totally accurate reflection. In the future, additional spatial scRNA-Seq studies will be necessary to validate these findings.

**Figure 7:**
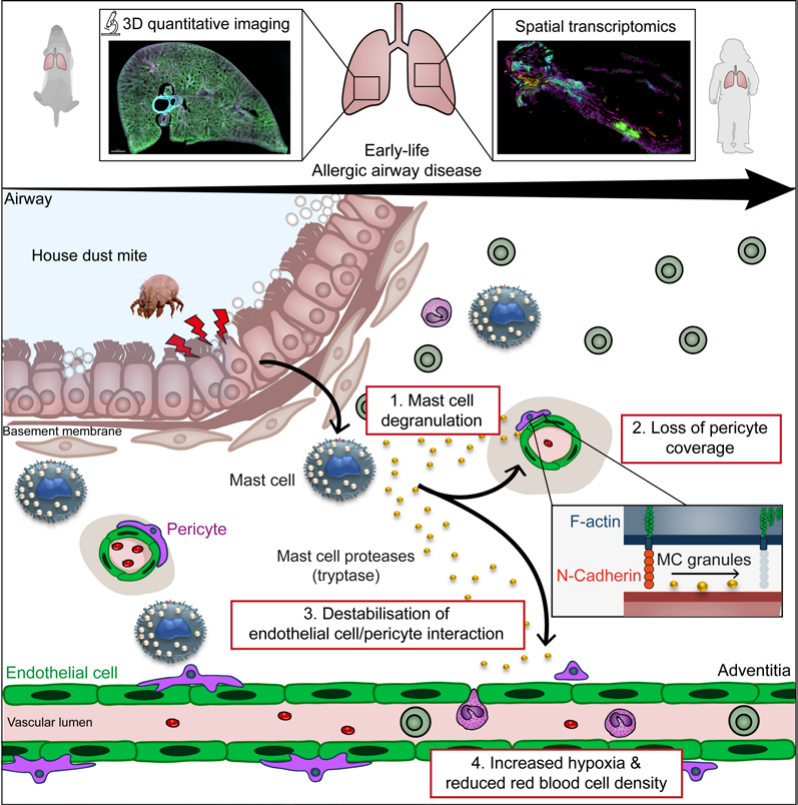
Model for the impact of MC activation on lung vasculature. MC degranulation during early life AAD leads to pericyte damage and associated vascular remodeling in the lung adventitia.

In summary, our study demonstrates that tissue remodeling, induced by inhaled allergen, affects blood vessel organization during early life with a reduction of the pulmonary microcirculation that precedes other structural changes. This loss of blood vessels in specific areas may negatively affect gas exchange. The need for increased oxygen supply due to tissue expansion and the concurrent reduction in pulmonary vasculature will lead to a quicker deterioration of lung and vascular function overall. Moreover, our study increases our fundamental understanding of temporal and regional responses during lung pathologies. Finally, we uncovered an axis of regulation for tissue remodeling during allergic inflammation involving MCs and pericytes that requires further exploration during respiratory disorders (Figure 7).

## Methods

### Sex as a biological variable.

Neonatal mice were of either sex, and litters were randomly assigned to control or experimental groups. Similar findings are reported for both sexes.

### Antibodies.

Anti-mouse CD140b (catalog 136002, RRID: AB_1953332), APC anti-mouse CD140b antibody (catalog 136008, RRID: AB_2268091), Alexa Fluor 647 anti-human CD31 antibody (catalog 303111, RRID:AB_493077), Alexa Fluor 488 anti-human CD31 antibody (catalog 303109, RRID:AB_493075), APC anti-human CD140b (PDGFRβ) antibody (catalog 323608, RRID:AB_2162787), Brilliant Violet 421 anti-mouse CD45 antibody (catalog 103133, RRID:AB_10001045), BV421 anti-human CD117 (C-kit) antibody (catalog 313215, RRID:AB_10896056), BV421 anti-mouse CD117 (catalog 560557, RRID:AB_1645258), PE/Cy5 anti-human CD45 (catalog 304010, RRID:AB_314398), APC anti-mouse FCεRIα (catalog 134316, RRID:AB_10640121), Brilliant Violet 711 anti-human TNF-α antibody (catalog 502940, RRID AB_2563885), and APC anti-mouse TER-119/erythroid cells antibody (catalog 116212 RRID AB_313713) were from BioLegend. Mouse/rat CD31/PECAM-1 antibody (catalog AF3628, RRID:AB_2161028), mouse mast cell protease-6/Mcpt6 antibody (catalog MAB3736, RRID:AB_2240825), and BV510 anti-human FCεRIα (catalog 334626, RRID:AB_2564291) were from R&D Systems

### Animals.

WT BALB/c mice (stock number 000651) were initially obtained from Charles River and maintained by in-house breeding. Each mother with its litter was housed separately. Mice were maintained in specific pathogen–free conditions and given food and water ad libitum. In individual experiments, all mice were matched for age and background strain.

### Human donors.

Human adult samples used in this research project were obtained from the Imperial College Healthcare Tissue Bank (ICHTB). ICHTB is supported by the National Institute for Health Research (NIHR) Biomedical Research Centre based at Imperial College Healthcare NHS Trust and Imperial College London. ICHTB is approved by Wales REC3 to release human material for research (17/WA/0161), and the samples for this project (R22006) were issued from subcollection reference number ICB_NC_21_017. School-age children (ages 8–17) who were undergoing a clinically indicated bronchoscopy were recruited. Donor clinical data are available in Supplemental Table 1.

### Neonatal AAD.

Neonatal mice were exposed repeatedly to either HDM (Greer Lot 360923) or PBS, intranasally from P7. During the first 2 weeks of life, mice were administered 10 μg of HDM extract in 10 μl of PBS 3 times a week. From week 3, mice received 15 μg of HDM in 15 μl of PBS. All outputs were assessed at 24 hours after allergen challenge (57).

### PCLSs.

PCLSs provided a 3D cell culture model to image vascular remodeling within the lung microenvironment and were adapted from previously described protocols (22, 58). For mouse PLCSs, lungs were inflated in situ with 0.4 ml of 2% low-melting agarose (Thermo Fisher Scientific) in PBS. After inflation, lungs were carefully dissected out and fixed in 4% paraformaldehyde (PFA) (Electron Microscopy Sciences) overnight at 4°C. Human lung tissue was collected from anonymized donors at Hammersmith and Royal Brompton Hospitals NHS Trusts and immediately fixed in 4% PFA overnight at 4°C. Following fixation, 100–200 μm transverse sections were prepared using a Compresstome VF-300 Vibrating Microtome (Precisionary Instruments).

PCLS were permeabilized in PBS complemented with 0.5% Triton (MilliporeSigma) for 1 hour at room temperature, then blocked in animal-free blocker (2BScientific Ltd.) for 1 hour. Slices were incubated with indicated primary antibodies overnight at 4°C in 25% animal-free blocker in PBS, and where required, PCLS were incubated with secondary antibodies for 5 hours at room temperature in 25% animal-free blocker in PBS. Lung slices were mounted on microscopic slides (Thermo Fisher), immersed in ProLong Diamond (Thermo Fisher), and kept at 4°C until image acquisition.

### Image acquisition.

Images were acquired with a Leica SP4 or SP8 using a ×20 objective (NA 0.7 and 0.75 for SP4 and SP8, respectively) or ×10 objective (NA 0.4 for SP4 and SP8) with a resolution of 512 × 512 or 1024 × 1024 pixels. Motorized stages of the microscopes were employed for tile-scan imaging and merged using Leica built-in software, version 5.1.0 (LAS) with a 10% overlap threshold. Adventitial regions (bronchovascular region) were defined as a lung region with the presence of a large airway (i.e., distinct stratified epithelium) and intermediate/large blood vessels. Vasculature associated to the adventitial region was analyzed within a 150 μm radius from the main airway/large vessel. Parenchymal regions were identified morphologically based on the alveoli structure more than 300 μm from any large airways.

### Image analysis.

Image analysis and rendering were performed using Imaris 8.1 or 9.3 (Bitplane). LIF files were converted into Imaris.ims files using Imaris Converter software, version 9.9.1 (Bitplane). Cell, HIF-1α, avidin^+^ granules, and m-MCP6^+^ spot number were analyzed using the semiautomatic spot function (cell diameter 10 and 15 μm for leukocytes and pericytes respectively; spot size 1–5 μm for vesicles and granules), and volumes (i.e., EC/pericyte coverage) were determined using the surface function. Numbers of cells were normalized to the total volume of the image. Thresholds for cell volume analysis in PCLS sections were maintained across the experimental conditions to provide accurate comparisons. Pericyte and endothelial cell volumes were determined using the cell function of Imaris, and similar thresholds for cell cytoplasm were used across all experimental conditions using either CMTMR staining or F-actin. All image analysis was performed on raw images without fluorescence modification.

### Cell purification.

Lung tissues were gently dissociated in small fragments (1 to 5 mm length) using scissors, then placed in 6-well plates (Corning). To generate pericytes, mouse and human lung samples were differentiated as previously described with small modifications (59). Briefly, cells were grown in DMEM supplemented with 10% FCS, 100 U/mL penicillin, 100 mg/mL streptomycin (all Thermo Fisher Scientific), and 100 pM pigment epithelium-derived factor (PEDF) (Sigma-Aldrich). After 15 to 22 days of culture, cells were detached using trypsin (Thermo Fisher) and isolated using the EasySep APC Positive Selection Kit II (STEMCELL Technologies) according to the manufacturer’s instructions. APC-conjugated anti-PDGFRβ was used for magnetic cell sorting. Cell purity (PDGFRβ^+^NG2^-^) was assessed by flow cytometry. Of note, human pericytes were smaller compared with mouse pericytes.

Mouse lung MCs were grown in OPTI-MEM supplemented with 10% FCS, 100 U/mL penicillin, 100 mg/mL streptomycin, and 4% CHO transfectants secreting murine stem cell factor (SCF) (a gift from P. Dubreuil, Centre de Recherche en Cancérologie de Marseille [CRCM], CNRS, INSERM, Aix-Marseille Univ, Institut Paoli-Calmettes, Marseille, France; 4% correspond to approximately 50 ng/ml SCF) for 6 to 8 weeks. Human lung MCs were grown in OPTI-MEM supplemented with 2.5% BSA, 100 U/mL penicillin, 100 mg/mL streptomycin, and 4% CHO transfectants secreting murine SCF for approximately 8 weeks. MC purity was assessed by flow cytometry (CD117^+^/FcεRI^+^).

### Functional assays.

Mouse or human pericytes (approximately 10,000 cells) were seeded onto μ-Slide 8 Well High ibiTreat, tissue culture treated (Ibidi), and left to rest for 24 hours. Cells were stained using CellTracker Orange CMTMR Dye (Thermo Fisher) according to the manufacturer’s instructions. Approximately 5,000 previously anti-DNP IgE-sensitized lung MCs (overnight at 1 μg/ml, clone SPE-7, Merck) were washed in Tyrode buffer (Merck) and placed on top of pericytes for approximately 15 minutes at 37°C. Increasing concentrations of DNP-BSA (Merck) were added, and cells were left for 24 hours at 37°C. In some experiments, protease inhibitor cocktail (Merck) was used according to the manufacturer’s instructions or pericytes were directly exposed to recombinant m-MCP6 (R&D Systems, matured according to the manufacturer’s instructions) or 10 μM of APC-366 (tryptase inhibitor, Merck) was employed to block human tryptase. Following incubation, cells were placed on ice and stained for surface markers for 30 minutes, washed, and fixed for approximately 15 minutes with 4% PFA at 37°C. Where necessary, fixed cells were permeabilized using permeabilization buffer (Thermo Fisher) and stained for 30 minutes at room temperature with fluorochrome-conjugated antibodies or for F-actin (Alexa Fluor 488 phalloidin, Thermo Fisher). Cells were kept in PBS before image acquisition. MC degranulation flow cytometry assays were performed as previously described (31). Briefly, approximately 10,000 IgE sensitized lung MCs were washed in Tyrode buffer (Merck) and placed in a 96-well U-bottom plates at 37°C. Cells were stimulated with increasing DNP-BSA concentrations (Merck) for 30 minutes at 37°C, collected, and processed for flow cytometry.

### Flow cytometry.

Cells were resuspended and washed in PBS + 0.5% BSA + 0.1 mM EDTA. Primary antibodies were incubated for approximately 30 minutes at 4°C, washed 2 times, and incubated with secondary antibodies if necessary for another 30 minutes at 4°C. Data were acquired with a BD LSR Fortessa using FACSDiva software (both from BD Biosciences) and analyzed using FlowJo software (version 10).

### scRNA-Seq analysis.

Mouse and human pericyte marker analysis was performed using the publicly available data-mining website the Lung Endothelial Cell Atlas (24). Data were visualized using the multigene query options, and the expression of the best 8 markers for pericytes was analyzed in mouse and human scRNA-Seq data sets (30,000 cells combined from 5 cohorts).

### Spatial transcriptomics.

We followed experimental methods (NanoString) described previously (37). Briefly, FFPE human lung samples were baked overnight at 37°C followed by 3 hours of baking at 65°C, then loaded onto a Leica Bond RX Fully Automated Research Stainer for subsequent processing steps. The processing protocol included 3 major steps: (a) slide baking, (b) antigen retrieval for 20 minutes at 100°C, and (c) treatment with proteinase K (1.0 μg/mL in 1× PBS) for 15 minutes. Following these steps, slides were removed from the Leica Bond RX, and a cocktail of GeoMx CTA probes (specific to approximately 1,800 gene separate targets) were applied to each slide and allowed to hybridize at 37°C overnight in a humidity chamber. The following day, slides were washed, blocked, and allowed to incubate with a combination of Alexa Fluor 488–labeled anti–α-SMA antibody (Invitrogen/Thermo product 53-9760-82; clone 1A4), Alexa Fluor 594–labeled anti-vimentin antibody (Santa Cruz Biotechnology Inc., sc-373717 AF594; clone E-5), Alexa Fluor 647–labeled anti-CD45 antibody (Cell Signaling Technology 13917BF; clone D9M8I), and Syto83 nucleic acid stain. Slides were stained for 1 hour at room temperature in a humidity chamber. Slides were then washed and loaded onto a GeoMx instrument. On the GeoMx machine, slides were fluorescently scanned, and ROIs were collected from the following areas: smooth muscle, epithelium, fibroblast (vessel rich), and immune rich. The GeoMx device exposed ROIs to 385 nm light (UV), releasing the indexing oligos. Indexing oligos were collected with a microcapillary and deposited into a 96-well plate. Samples were dried down overnight, then resuspended in 10 μL of DEPC-treated water. PCR was performed using 4 μL of each sample, and the oligos from each ROI were indexed using unique i5 and i7 dual-indexing systems (Illumina). PCR reactions were purified twice using AMPure XP beads (Beckman Coulter Inc.) according to the manufacturer’s protocol. Purified libraries were sequenced on an Illumina NovaSeq 6000. Data analysis was performed as described (37). Following removal of targets consistently below the limit of quantification (i.e., <5,000 raw reads) and negative probes, the limit of detection above which a gene was called detected was defined as 2 SD above the geometric mean of negative probes. Data sets were normalized using upper quartile (Q3) normalization. Data analysis was then performed using the DSP platform and R software. Cell deconvolution analysis was performed using the following R packages: GSVA, Stringr, and Dplyr (60). Original scRNA-Seq data for cell deconvolution were obtained from Deprez et al. (38).

### Statistics.

Neonatal mice were randomly assigned to control or experimental groups. The number of mice analyzed for each different experimental approach is indicated on each figure. Most statistical analyses, including PCA, were performed using Prism software (GraphPad, version 9.4.1). Pathway analysis (GSEA) was performed on the DSP platform using a Mann-Whitney test corrected for multiple testing using the Benjamini-Hochberg (BH) procedure, and data were represented using Prism software (version 10.1.2). t-SNE analysis was performed using build-in functions of FlowJo software (version 10.8.1); iterations were fixed at 1,000, perplexity 30, and learning rate 22. Data are represented as means ± SEM, and *n* for each data set are provided in the figure legends. Comparisons between 2 groups were carried out using paired or unpaired Student’s or Mann-Whitney *t* test as appropriate. One-way ANOVA followed by Tukey’s post hoc test was performed for multiple group comparisons. Statistical significance was defined as *P* < 0.05.

### Study approval.

All in vivo experiments were conducted at the NHLI, Imperial College London, UK, under UK legislation for animal experimentation (PPL P996A24E1 and PP8328343) and approved according to the UK Home Office Animals Scientific Procedures Act 1986 (ASPA) and the Imperial College’s Animal Welfare and Ethical Review Body (AWERB) at Imperial College, London. Human studies were approved by the institutional ethics committee, and written, informed parental consent and child assent were obtained.

### Data and code availability.

Values for all data points in graphs are reported in the Supporting Data Values file. Spatial transcriptomic data are available as Supplemental Data Sets 1, 2, and 3. Any additional information required to reanalyze the data reported, such as original source images in this paper, is available upon request. The paper does not report original code.

## Author contributions

RJ, FP, and CML conceived the project. RJ and FP created the methodology to analyze experiments. RJ, FP, HS, AV, MGM, and LJE performed experiments. WJT, MA, KB, ES, PLM, RJH, SAW, LY, and SS provided resources. RJ, FP, and AV performed formal analysis. RJ created all the figures. RJ wrote the original draft of the manuscript. RJ, FP, HS, WJT, AV, LJE, MGM, MA, KB, ES, PLM, RJH, SAW, LY, SS,and CML reviewed and edited the manuscript. RJ and CML supervised the project.

## Supplementary Material

Supplemental data

Supplemental data set 1

Supplemental data set 2

Supplemental data set 3

Supplemental table 1

Supplemental table 2

Supplemental table 3

Supplemental table 4

Supplemental video 1

Supplemental video 2

Supplemental video 3

Supporting data values

## Version 1 03/15/2024

Electronic publication
